# Effects of preoperative and postoperative resistance exercise interventions on recovery of physical function in patients undergoing abdominal surgery for cancer: a systematic review of randomised controlled trials

**DOI:** 10.1136/bmjsem-2017-000331

**Published:** 2018-04-25

**Authors:** David Stephensen, Ferhana Hashem, Kevin Corbett, Amanda Bates, Michelle George, Ralph Peter Hobbs, Malcolm Hopkins, Irena Hutchins, David Peter Lowery, Tracy Pellatt-Higgins, Charitini Stavropoulou, Ian Swaine, Lee Tomlinson, Hazel Woodward, Haythem Ali

**Affiliations:** 1 Physiotherapy Department, East Kent Hospitals University Foundation NHS Trust, Canterbury, UK; 2 Centre for Health Service Studies, University of Kent, Canterbury, UK; 3 Centre for Critical Research in Nursing and Midwifery, Middlesex University, London, UK; 4 Research and Development, Maidstone and Tunbridge Wells NHS Trust, Maidstone, UK; 5 School of Health Sciences, City University London, London, UK; 6 Centre for Science and Medicine in Sport and Exercise, University of Greenwich, Chatham, UK

**Keywords:** exercise training, cancer, abdomen, surgery, physical fitness

## Abstract

**Objective:**

To systematically review the effects of preoperative and postoperative resistance exercise training on the recovery of physical function in patients undergoing abdominal surgery for cancer.

**Data sources:**

A systematic review of English articles using Medline, Physiotherapy Evidence Database, CINAHL and the Cochrane Library electronic databases was undertaken.

**Eligibility criteria for selecting studies:**

Studies were included if they used a randomised, quasi-randomised or controlled trial study design and compared the effects of a muscle-strengthening exercise intervention (±other therapy) with a comparative non-exercise group; involved adult participants (≥18 years) who had elected to undergo abdominal surgery for cancer; and used muscle strength, physical function, self-reported functional ability, range of motion and/or a performance-based test as an outcome measure.

**Results:**

Following screening of titles and abstracts of the 588 publications retrieved from the initial search, 24 studies met the inclusion criteria and were accessed for review of the full-text version of the article, and 2 eligible studies met the inclusion criteria and were included in the review. One exercise programme was undertaken preoperatively and the other postoperatively, until discharge from hospital. The exercise interventions of the included studies were performed for five and eight sessions, respectively. There were no differences between groups in either study.

**Conclusion:**

The only two studies designed to determine whether preoperative or postoperative resistance muscle-strengthening exercise programmes improved or negatively affected physical function outcomes in patients undergoing abdominal surgery for cancer provide inconclusive results.

**What is already known?**

- Abdominal and thoracic cancers cause debilitating illness, and surgery is associated with significant decline in physical function.
- Exercise initiated after completion of active cancer treatment has a beneficial effect on health-related quality of life.

**What are the new findings?**

- There is insufficient evidence that preoperative or postoperative resistance muscle-strengthening exercise improves or negatively affects functional outcomes in patients undergoing abdominal surgery for cancer.
- Large-scale, well-designed clinical trials are required to determine whether resistance muscle-strengthening exercise is beneficial for patients undergoing abdominal surgery for cancer.

## Introduction

### Background

Abdominal and thoracic cancers affect about 12 000 people annually in the UK. Many of these patients will undergo surgery, after which there is a high risk of postoperative complications and significant decline in physical function. A systematic review of exercise for people with cancer by Stevinson *et al*^1^ found some evidence that those who exercised had better physical function compared with those who did not exercise, but there was insufficient evidence to demonstrate improvement in quality of life. In addition, they were not able to determine which type of exercise intervention was best or if any had long-term benefit. A more recent Cochrane review of exercise for people with cancer by Mishra *et al*^2^ found that exercise initiated after completion of active cancer treatment (ie, surgery, chemotherapy, radiation therapy or hormone therapy) has a beneficial effect on health-related quality of life, although no parallel improvement in self-reported physical function was found. The exercise interventions included in this review varied greatly and included strength training, yoga, walking, cycling, tai chi and qi gong. However, due to the small number of studies available, these authors were not able to evaluate the effect of different modes and intensities of exercise. Furthermore, studies of exercise in the preoperative and early postoperative stages were not included in the review. Therefore, it is not known whether exercise, when commenced before the end of active cancer treatment, would have additional benefit on physical function for those undergoing surgery.

While there is growing evidence on the beneficial effects of aerobic exercise, resistance exercise training has received much less attention.^3–6^ It is thought that resistance exercise training could act to aid recovery of muscle function.^7^ It has long been established that resistance exercise training is effective in stimulating muscle anabolic processes and increasing muscle strength.^8^ It may even counteract some of the metabolic pathophysiology associated with cachexia.^9^ Furthermore, it can be performed with very little equipment and space and while patients are bed-bound in hospital or at home. Although there have been previous systematic reviews of the effects of exercise training, there have not been any that have specifically focused on resistance training.

Previous reviews, relating to exercise training for patients with cancer, have mostly focused on specific outcomes such as fatigue and quality of life,^4 5^ and most have centred on specific types of cancer.^10–17^ Galvão and Newton^18^﻿ published a review of exercise intervention studies for all cancers and a meta-analysis of exercise training interventions. However, their review included a heterogeneous group of studies including some that were not randomised or had no control group. Quality systematic reviews require critical appraisal of the quality of the reviewed studies and share accurate descriptions of the design, delivery and interpretation of what was done in the study. In some instances detailed description of these aspects is not available.^19^

One of the main challenges in studying the effects of a resistance exercise programme on physical function in cancer surgery patients is in identifying an appropriate outcome measure. The review by Mishra and colleagues found no significant improvement in physical function as evaluated using self-report questionnaires, but they did not measure any index of physical performance.^2^ Therefore, our aim was to undertake a systematic review of the literature on interventional studies investigating the effects of preoperative and postoperative resistance exercise training on recovery of physical function in patients undergoing abdominal surgery for cancer. The findings will provide clinicians and investigators a basis to choose exercise interventions for use in clinical practice or for future research.

## Methods

**Figure 1:**
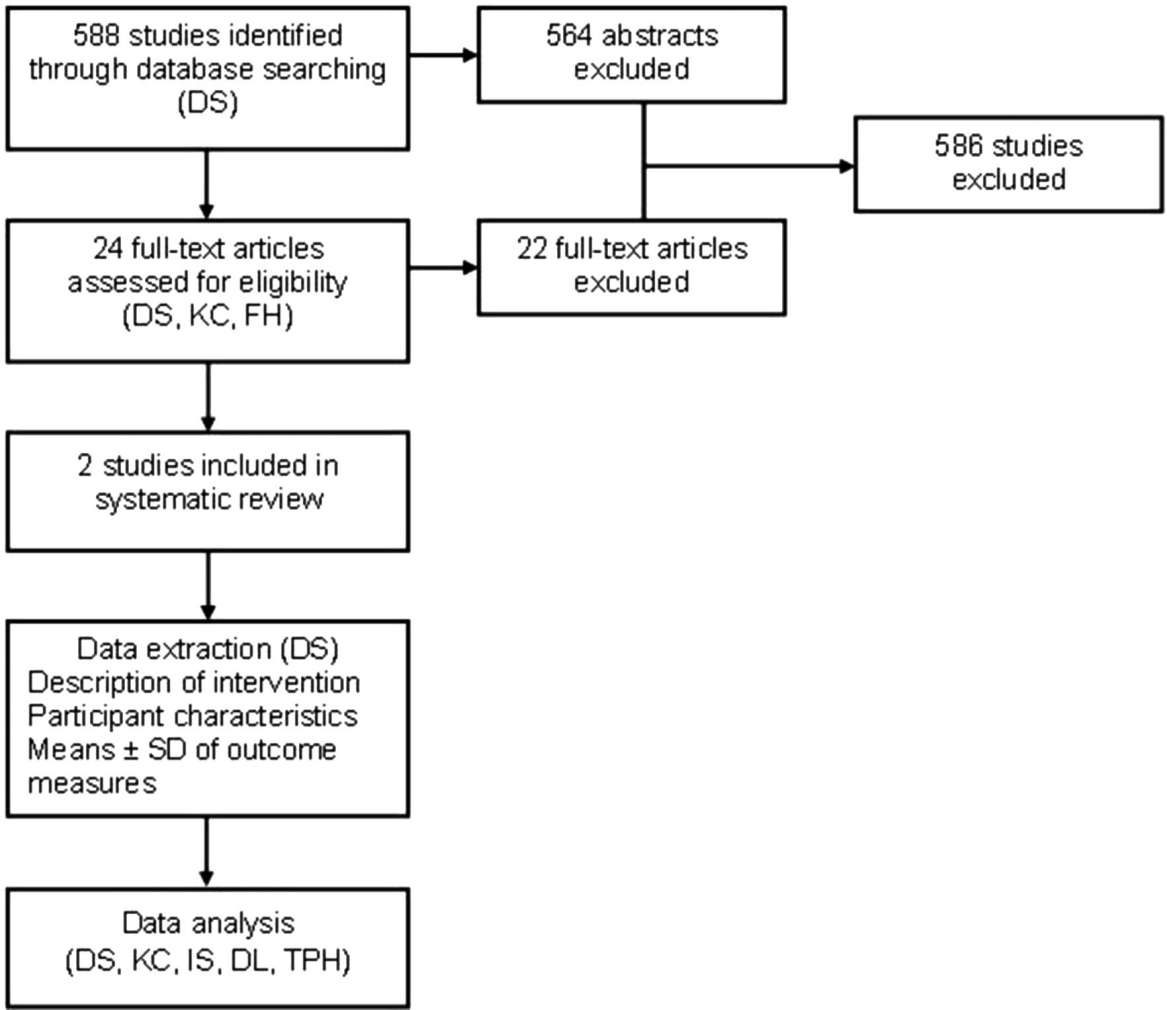
Flow chart for systematic review of studies.

The Preferred Reporting Items for Systematic Reviews and Meta-Analyses guidelines on systematic reviews were followed for this review.^20^ Figure 1 summarises the review process.

### Search strategy

The Cochrane Library, EBSCO (SPORTDiscus and Cumulative Index to Nursing and Allied Health Literature (CINAHL)), PLOS, PubMed (Medline) and Elsevier (Scopus) electronic databases were searched up to and including December 2014. The search strategy used was exercise OR training OR isometric OR static OR isotonic OR concentric OR eccentric OR resistance OR strengthening exercise OR exercise therapy OR circuit training OR rehabilitation OR physiotherapy; AND neoplasm OR abdominal cancer OR stomach cancer OR gastric cancer OR bowel cancer OR pancreatic cancer OR colorectal cancer OR colon cancer OR rectal cancer OR gastrointestinal cancer OR ovarian cancer OR endometrial cancer OR cervical cancer OR renal cancer OR kidney cancer OR bladder cancer OR uterine cancer OR gynaecological cancer OR urological cancer; AND abdominal surgery OR laparotomy OR laparoscopy OR laparoscopic OR anterior resection OR colectomy OR hemicolectomy; AND clinical trial OR random controlled trial OR quasi-randomised controlled trial OR controlled trial OR comparative trial.

All titles and abstracts generated by the search were independently screened for inclusion by three authors (DS, FH and KC). Disagreement between authors was discussed and consensus was reached. The search was restricted to English language and were included if the following criteria were met: (1) randomised, quasi-randomised or controlled trial study design comparing a muscle-strengthening exercise intervention (ie, exercise using resistance to induce muscular contraction) ± other therapy with a comparative group; (2) included adult participants (≥18 years) who underwent abdominal surgery (ie, surgery pertaining to the contents of the abdominal cavity, its walls and orifices) for cancer; and (3) included muscle strength, physical function, self-reported functional ability, range of motion and/or performance-based test as an outcome measure.

### Data extraction

Participants’ age, gender, diagnosis, surgical procedure and sample size were extracted from the included studies, along with a description of the exercise intervention, including muscle group or groups exercised, contraction effort, number of repetitions and frequency, length of programme, length of follow-up, group or individual exercise programme, home or supervised exercise programme, and timing of programme (presurgery and/or postsurgery).

### Data synthesis and analysis

The aim of this review was to evaluate the effect of resistance muscle strengthening on physical function in people undergoing abdominal surgery for cancer. For each study, means and SD of outcomes focused on physical function were extracted. Outcomes relating directly to surgery, length of stay, infection and other postsurgical complications were not considered in this review.

Assessment was made of the outcome measures for physical function that were used in different studies, before progression to pooling of data for analysis of the most common outcome measure. Treatment effect of individual studies is reported as mean difference and 95% CIs, and the data summarised.

Risk of bias was assessed with the Physiotherapy Evidence Database (PEDro) scale.^21^ Items assessed included exclusion criteria, procedures for group allocation and missing data, participant, therapist and assessor blinding, and reporting of results. Studies were then graded using the Cochrane Reviews Grading of Recommendations Assessment, Development and Evaluation criteria.^21 22^

## Results

### Search strategy and selection of articles

The initial search strategy resulted in 588 publications. Following screening of titles and abstracts, 24 studies met the inclusion criteria and were accessed for review of the full text, of which 2 eligible studies^23 24^ were included in the review (see table 1 and figure 1). Full-text studies were excluded for a number of reasons: (1) the study lacked a well-defined muscle-strengthening intervention (n=18); (2) the study did not include patients undergoing abdominal surgery for cancer (n=4); and (3) the study did not use a physical function outcome measure (muscle strength, self-report questionnaires or physical performance measures).

**Table 1:** Characteristics of included studies

	Methods	Participants	Intervention	Relevant outcomes	Risk of bias
Dronkers *et al*^23^	Randomised study investigating the preoperative effect of an exercise programme in participants with colon cancer.	Exercise group, n=22Age: 71.1±6.3Gender: 15 male, 7 femaleControl group, n=20Age: 68.8±6.4Gender: 16 male, 4 female	Supervised programme 2×week for 2–4 weeks (mean 5.1±1.9) and home-based programme of walking or cycling for a minimum of 30 min per day (perceived exertion of 11–13 Borg Scale). Programme: Warm up. Resistance training of the lower limb extensors—equipment and method not stated (maximum of 1 set of 8–15 repetitions at 60%–80% of the one repetition maximum). Inspiratory muscle training (10%–60% max inspiratory pressure for 240 breathing cycles. Aerobic training—method and equipment not stated (55%–75% max HR or perceived exertion of 11–13 Borg Scale for 20–30 min). Functional activities according to patients’ capabilities and interests (Vreede *et al*,^28^ regimen—no other information provided).	Timed Up and GoChair rise timePhysical Activity QuestionnaireAbbreviated Fatigue QuestionnaireEORTC QLQ-C30 Global Health/Functional Scale/Symptom Scale	PEDro score 8/11GRADE criteria—moderate
Ahn *et al*^24^	Randomised study investigating the effect of a postsurgical, inpatient exercise programme in patients with stages I–III colon cancer.	Exercise group, n=17Age: 55.61±7.11Gender: 12 male, 5 femaleControl group, n=14Age: 57.43±6.12Gender: 5 male, 9 female	Supervised exercise programme 2×day, 15 min/sessionSubdivided into three phases: Implemented while subjects were still unable to get out of bed: stretching (neck, shoulder, wrist, ankle and pelvis), pelvic tilt—isometric, resistance exercise (ankle dorsiflexion and plantar flexion against the hand of the therapist), unsupervised sitting or walking in the ward. Performed once subjects were able to get out of the bed, but had limited ambulation: stretching (whole body, leg and shoulder), pelvic tilt and thrust, one leg raise, crunch, resistance exercise (1 set, 10 repetitions) with 1 lb weight (chest, shoulder, arm, thigh and calf), unsupervised walking. Performed when subjects were able to ambulate without any discomfort; in addition to phase 2 exercises, resistance strengthening increased to 12 repetition×3 sets, supervised balance exercises—one leg standing, one leg calf raise, hip adduction, hip abduction, hip flexion with knee bent, hip extension, unsupervised walking.	Timed one-leg standSit-to-stand in 30 sTecumseh step test	PEDro score 8/11GRADE criteria—moderate

EORTC QLQ-C30, European Organization for Research and Treatment of Cancer Quality of Life Questionnaire; GRADE, Grading for Recommendations Assessment, Development and Evaluation; HR, heart rate; PEDro, Physiotherapy Evidence Database.

### Description of included studies

Characteristics of the participants and intervention of the two included studies are presented in table 1. Both were small (n=42 and 31) single-centre studies investigating participants undergoing abdominal surgery for excision of cancer of the colon. Dronkers *et al*^23^ investigated the effect of a preoperative exercise programme on preoperative outcomes, and Ahn *et al*^24^ investigated the effect of a postoperative exercise programme on short-term outcomes at discharge from hospital. The participants in the preoperative study were aged 10–15 years older than those in the postoperative study. In terms of gender, a higher proportion of men participated in both studies.

The preoperative intervention of Dronkers *et al*^23^ included a twice-weekly supervised exercise programme and a home-based programme of walking or cycling for a minimum of 30 min per day for 2–4 weeks before admission for surgery. In addition to a single set of resistance strengthening exercises of the leg (8–15 repetitions at 60%–80% of the one repetition maximum), the programme included inspiratory muscle training, aerobic training at 55%–75% max heart rate (HR) or perceived exertion of 11–13 Borg Scale for 20–30 min, and functional activities. A full description of the resistance exercise was not published. Three of the intervention groups (13.6%) did not complete the study with their data analysed as intention to treat.

The postoperative intervention of Ahn *et al*^24^ comprised a twice-daily 15 min supervised exercise programme performed by the participant until discharge from hospital (mean 8.87±2.28 days). In addition to resistance strengthening exercises of the chest, shoulder, arm, thigh and calf leg, the programme included stretching exercises for the neck, shoulder, wrist, ankle and pelvis, core trunk exercises and ambulation. In terms of the strengthening exercises, resistance was applied manually by the therapist initially and then using 1 lb free weights. During phase 2, one set of 10 repetitions was performed, and in phase 3, three sets of 12 repetitions were performed. Because these studies used different outcome measures, it was not possible to pool the data in order to analyse mean changes in physical function outcomes.

### Risk of bias of included studies

The methodological quality of the two included studies was rated as moderate according to the GRADE criteria, that is, randomised studies with unclear bias or well-designed observational studies with large, consistent and precise estimates of the magnitude of an intervention effect. Difficulty in blinding trial participants and therapists to the intervention meant studies were not rated as high. Both studies scored 8 out of 11 on the PEDro scale. Block randomisation using prepared envelopes, stratified by age (60–70 and >70) by someone independent of the study, was used in the preoperative study. Randomisation, at a 1-to-1 ratio, into study groups via minimisation to balance prognostic factors between groups (age and gender) was used in the postoperative study. In the preoperative study the gender distribution was similar in the control and intervention groups; however, in the postoperative study, twice as many men were randomised to the exercise group than the control group despite the minimisation procedures to balance gender between groups. In relation to the description of the intervention, some information was lacking in terms of equipment and methodology with regard to the aerobic and functional activity components of the preoperative intervention.

### Effect of strengthening exercise

#### Preoperative muscle strengthening

The mean difference and upper and lower 95% CI between the control and intervention group in the study by Dronkers *et al*^23^ are shown in table 2. The five-session preoperative exercise programme had no significant effect on preoperative Timed Up and Go, chair rise time test, self-reported physical activity, quality of life and fatigue. Statistical power for six out of the seven measures was unacceptably low. Effect on postsurgery outcomes was not evaluated.

**Table 2:** Summary of effect of exercise intervention

	Mean between-group difference	Lower 95% CI	Upper 95% CI	Statistical power*
Dronkers *et al*,^23^﻿ preoperative intervention				
Timed Up and Go (s)	−1.20	−2.78	0.38	31.2
Chair rise (s)	−5.40	−9.24	−1.56	77.3
Physical activity (min/day)	44.00	−141.82	229.82	7.3
Abbreviated Fatigue Questionnaire	−3.90	−7.41	−0.39	57.6
EORTC QLQ-C30 (Global Health)	−4.00	−15.57	7.57	10.2
EORTC QLQ-C30 (Functional Scale)	12.00	−28.26	52.86	87.4
EORTC QLQ-C30 (Symptom Scale)	36.00	−31.09	103.09	17.7
Ahn *et al*,^24^ ﻿ postoperative intervention				
Timed one-leg stand (s)	−7.28	−16.25	1.69	40.0
Sit-to-stand (repetitions)	−2.00	−5.78	1.78	17.7
Tecumseh step test (heart rate, beats/min)	10.29	1.63	18.95	64.8

*Probability of rejecting a false null hypothesis (where α=0.05), for a between-group comparison of means at study endpoint.

#### Postoperative muscle strengthening

The mean difference and upper and lower 95% CI between the control and intervention groups in the study by Ahn *et al*^24^ are also shown in table 2. The inpatient postoperative exercise programme had no significant effect at time of discharge from hospital on ability to balance on one leg, number of sit-to-stands in 30 s or aerobic capacity (estimated from performance of the Tecumseh step test). Statistical power was not sufficient to allow any conclusion for or against the preferential use of any of the outcome measures that were used in this trial. Effect on functional recovery postdischarge from hospital was not evaluated.

## Discussion

Our aim was to systematically review the evidence on the effectiveness of preoperative and postoperative strengthening exercises on short-term and long-term recovery of physical function in patients undergoing abdominal surgery for cancer. Two studies were included, which represented 73 patients (48 men and 25 women) undergoing abdominal surgery for cancer. One exercise programme was undertaken preoperatively and the other postoperatively until discharge from hospital. This represents insufficient evidence to determine whether this type of preoperative or postoperative resistance muscle-strengthening exercise programme improves or negatively affects functional outcomes in patients undergoing abdominal surgery for cancer.

The study by Dronkers *et al*,^23^ which investigated a preoperative exercise programme, was statistically underpowered with the exception of the functional measure derived from the quality of life scale. The programme included resistance strengthening of the lower limb muscle extensors and was performed for a mean of five sessions. This may not be sufficient to provide an adequate training stimulus to significantly increase muscle strength. Indeed, guidelines published by the American College of Sports Medicine recommend resistance exercise 2–3 times per week with 2–4 sets of 10–15 repetitions to improve strength in middle-aged and older persons.^25^

In contrast, the study by Ahn *et al*^24^ investigated a postoperative exercise programme, but this was also statistically underpowered and provides inconclusive evidence in support of the intervention and the use of particular outcome measures. The intervention was different from that of Dronkers *et al*^23^ in that it used a progressive resistance programme involving the upper and lower limbs, together with stretching, functional balance strengthening and walking. Also, isometric strengthening exercises were commenced early postoperatively while the patient was still in bed and then progressed to ‘resistance-through-range’ strengthening as well as balance strengthening exercises, until discharge from hospital. The mean hospital length of stay, for the study of Dronkers *et al*^23^ was 7 days for the control group, and in the exercise group it was 8 days. Similarly, for the study by Ahn *et al*,^24^ it was 8 days of exercise, and it is likely that this will not provide an adequate training stimulus to significantly increase muscle strength and function.

There are some limitations to our review. We limited our inclusion by study design, only including randomised or quasi-randomised studies where there was a clear resistance muscle strengthening component as part of an exercise programme. It is possible that other studies have included muscle-strengthening exercises or functional exercises that will have an effect on muscle strength that have not been included in this review due to our inclusion criteria, and we advocate the Consensus on Exercise Reporting Template guidelines for reporting exercise intervention studies.^26^ The two studies included in the review recruited almost twice as many men as women, and the results may not reflect the general population. Future studies should focus on detailed descriptions of the exercise intervention, consistent outcome measures and longer intervention and follow-up times.^27^

Our systematic review suggests that the use of resistance exercise interventions for recovery of physical function in patients undergoing abdominal surgery for cancer must be considered with caution. The small number of included underpowered studies and the inability to pool the results due to the heterogeneity of outcome measures mean that there is a lack of evidence for or against the use of this type of resistance muscle-strengthening exercise programmes to improve functional outcomes in these patients. While the studies give encouraging preliminary evidence that muscle-strengthening programmes may be feasible for abdominal cancer surgery patients, further large-scale, well-designed clinical trials are required to determine whether this type of exercise intervention is beneficial for this group of patients.

## References

[1] Stevinson C, Lawlor DA, Fox KR Exercise interventions for cancer patients: systematic review of controlled trials. Cancer Causes Control 2004;15:1035–56. 10.1007/s10552-004-1325-4 15801488

[2] Mishra SI, Scherer RW, Geigle PM, et al Exercise interventions on health-related quality of life for cancer survivors. Cochrane Database Syst Rev 2012;8:CD007566 10.1002/14651858.CD007566.pub2 PMC7387117 22895961

[3] Pinto BM, Maruyama NC Exercise in the rehabilitation of breast cancer survivors. Psychooncology 1999;8:191–206. 10.1002/(SICI)1099-1611(199905/06)8:3<191::AID-PON355>3.0.CO;2-T 10390732

[4] Courneya KS Exercise in cancer survivors: an overview of research. Med Sci Sports Exerc 2003;35:1846–52. 10.1249/01.MSS.0000093622.41587.B6 14600549

[5] Dimeo FC Effects of exercise on cancer-related fatigue. Cancer 2001;92(6 Suppl):1689–93. 10.1002/1097-0142(20010915)92:6+<1689::AID-CNCR1498>3.0.CO;2-H 11598888

[6] Fairey AS, Courneya KS, Field CJ, et al Physical exercise and immune system function in cancer survivors: a comprehensive review and future directions. Cancer 2002;94:539–51. 10.1002/cncr.10244 11900239

[7] Komi PV, Viitasalo JT, Rauramaa R, et al Effect of isometric strength training of mechanical, electrical, and metabolic aspects of muscle function. Eur J Appl Physiol Occup Physiol 1978;40:45–55. 10.1007/BF00420988 569576

[8] Cheema B, Gaul CA, Lane K, et al Progressive resistance training in breast cancer: a systematic review of clinical trials. Breast Cancer Res Treat 2008;109:9–26. 10.1007/s10549-007-9638-0 17624588

[9] Lenk K, Schuler G, Adams V Skeletal muscle wasting in cachexia and sarcopenia: molecular pathophysiology and impact of exercise training. J Cachexia Sarcopenia Muscle 2010;1:9–21. 10.1007/s13539-010-0007-1 21475693 PMC3060644

[10] Schwartz AL Daily fatigue patterns and effect of exercise in women with breast cancer. Cancer Pract 2000;8:16–24. 10.1046/j.1523-5394.2000.81003.x 10732535

[11] Schwartz AL Fatigue mediates the effects of exercise on quality of life. Qual Life Res 1999;8:529–38. 10.1023/A:1008978611274 10548868

[12] Schwartz AL, Mori M, Gao R, et al Exercise reduces daily fatigue in women with breast cancer receiving chemotherapy. Med Sci Sports Exerc 2001;33:718–23. 10.1097/00005768-200105000-00006 11323538

[13] Courneya KS, Keats MR, Turner AR Physical exercise and quality of life in cancer patients following high dose chemotherapy and autologous bone marrow transplantation. Psychooncology 2000;9:127–36. 10.1002/(SICI)1099-1611(200003/04)9:2<127::AID-PON438>3.0.CO;2-L 10767750

[14] Dimeo F, Rumberger BG, Keul J Aerobic exercise as therapy for cancer fatigue. Med Sci Sports Exerc 1998;30:475–8. 10.1097/00005768-199804000-00001 9565925

[15] Courneya KS, Friedenreich CM Physical exercise and quality of life following cancer diagnosis: a literature review. Ann Behav Med 1999;21:171–9. 10.1007/BF02908298 10499138

[16] Courneya KS Exercise interventions during cancer treatment: biopsychosocial outcomes. Exerc Sport Sci Rev 2001;29:60–4. 10.1097/00003677-200104000-00004 11337824

[17] Friendenreich CM, Courneya KS Exercise as rehabilitation for cancer patients. Clin J Sport Med 1996;6:237–44. 10.1097/00042752-199610000-00006 8894336

[18] Galvão DA, Newton RU Review of exercise intervention studies in cancer patients. J Clin Oncol 2005;23:899–909. 10.1200/JCO.2005.06.085 15681536

[19] Weir A, Rabia S, Ardern C Trusting systematic reviews and meta-analyses: all that glitters is not gold! Br J Sports Med 2016;50:1100–1. 10.1136/bjsports-2015-095896 26968215

[20] Liberati A, Altman DG, Tetzlaff J, et al The PRISMA statement for reporting systematic reviews and meta-analyses of studies that evaluate healthcare interventions: explanation and elaboration. BMJ 2009;339:b2700 10.1136/bmj.b2700 19622552 PMC2714672

[21] Herbert R, Moseley A, Sherrington C PEDro: a database of randomised controlled trials in physiotherapy. Health Inf Manag 1998;28:186–8. 10.1177/183335839902800410 10387366

[22] Cochrane Handbook Chapter 11 (Presenting results and ‘Summary of findings’ tables). http://handbook.cochrane.org/chapter_11/11_presenting_results_and_summary_of_findings_tables.htm

[23] Dronkers JJ, Lamberts H, Reutelingsperger IM, et al Preoperative therapeutic programme for elderly patients scheduled for elective abdominal oncological surgery: a randomized controlled pilot study. Clin Rehabil 2010;24:614–22. 10.1177/0269215509358941 20530651

[24] Ahn KY, Hur H, Kim DH, et al The effects of inpatient exercise therapy on the length of hospital stay in stages I-III colon cancer patients: randomized controlled trial. Int J Colorectal Dis 2013;28:643–51. 10.1007/s00384-013-1665-1 23417645

[25] Garber CE, Blissmer B, Deschenes MR, et al Quantity and quality of exercise for developing and maintaining cardiorespiratory, musculoskeletal, and neuromotor fitness in apparently healthy adults: guidance for prescribing exercise. Med Sci Sports Exerc 2011;43:1334–59. 10.1249/MSS.0b013e318213fefb 21694556

[26] Slade SC, Dionne CE, Underwood M, et al Consensus on Exercise Reporting Template (CERT): explanation and elaboration statement. Br J Sports Med 2016;50:1428–37. 10.1136/bjsports-2016-096651 27707738

[27] Ardern CL Systematic review hacks for the sports and exercise clinician: five essential methodological elements. Br J Sports Med 2016;50:447–9. 10.1136/bjsports-2015-095548 26612842

[28] de Vreede PL, van Meeteren NL, Samson MM, et al The effect of functional tasks exercise and resistance exercise on health-related quality of life and physical activity. A randomised controlled trial. Gerontology 2007;53:12–20. 10.1159/000095387 16940735

